# Human mesenchymal stem cell-derived extracellular vesicles/estrogen combined therapy safely ameliorates experimentally induced intrauterine adhesions in a female rat model

**DOI:** 10.1186/s13287-018-0924-z

**Published:** 2018-06-28

**Authors:** Nesrine Ebrahim, Ola Mostafa, Rania Ebrahim El Dosoky, Inas A. Ahmed, Ahmed S. Saad, Abeer Mostafa, Dina Sabry, Khalid Abdelaziz Ibrahim, Ayman Samir Farid

**Affiliations:** 1 Department of Histology and Cell Biology, Faculty of Medicine, Benha University, Banha, 13518 Qalyubia Egypt; 2 Stem Cell Unit, Faculty of Medicine, Benha University, Banha, 13518 Qalyubia Egypt; 3 Department of Medical Biochemistry, Faculty of Medicine, Benha University, Banha, 13518 Qalyubia Egypt; 4 Molecular Biology and Biotechnology Unit, Faculty of Medicine, Benha University, Banha, 13518 Qalyubia Egypt; 5 Department of Obstetrics and Gynecology, Faculty of Medicine, Benha University, Banha, 13518 Qalyubia Egypt; 6 Department of Medical Biochemistry, Faculty of Medicine, Cairo University, Cairo, 11562 Egypt; 7 Molecular Biology and Stem Cell Unit, Faculty of Medicine, Cairo University, Cairo, 11562 Egypt; 8 Department of Clinical Pathology, Faculty of Veterinary Medicine, Benha University, Moshtohor, Toukh, 13736 Qalyubia Egypt

**Keywords:** Intrauterine adhesions, UCMSCs-EVs, Estrogen, TNF-α, TGF-β, IL-1, IL-6, RUNX2, Collagen

## Abstract

**Background:**

Mesenchymal stem cells (MSCs) have diverse functions in regulating injury and inflammation through the secretion of extracellular vesicles (EVs).

**Methods:**

In this study, we investigated the systemic administration of extracellular vesicles derived from human umbilical cord mesenchymal stem cells (UCMSCs-EVs) as a therapeutic agent for intrauterine adhesions (IUAs) caused by endometrial injury. Additionally, we investigated the therapeutic impact of both UCMSCs-EVs and estrogen either separately or in combination in a rat model. The inflammation, vascularization, proliferation, and extent of fibrosis were assessed by a histopathological and immunohistochemical assessment using transforming growth factor (TGF)-β as a fibrotic marker and vascular endothelial growth factor (VEGF) as a vascular marker. Additionally, quantitative real-time polymerase chain reaction (qRT-PCR) was used to analyze the expression of tumor necrosis factor (TNF)-α, interleukin (IL)-1, IL-6 (inflammatory cytokines), CD140b (a marker of endometrial stem cells), and RUNX2 (an antifibrotic factor). Finally, Western blotting was used to evaluate collagen I and β-actin expression.

**Results:**

The therapeutic groups treated with either UCMSCs-EVs alone or combined with estrogen exhibited a significant decrease in inflammation and fibrosis (TNF-α, TGF-β, IL-1, IL-6, RUNX2, and collagen-I) as well as a significant decrease in vascularization (VEGF) compared with the untreated rats with IUAs. The most significant results were obtained in animals with IUAs that received a combined therapy of UCMSCs-EVs and estrogen.

**Conclusions:**

We conclude that the synergistic action of human UCMSCs-EVs combined with estrogen provides a highly effective alternative regenerative agent in IUA treatment.

## Background

Intrauterine adhesion (IUA), which is caused by damage to the endometrial basement membrane and exposure of myometrial tissue, is a common gynecological disease that is also referred to as Asherman’s syndrome and is characterized by spanomenorrhea, amenorrhea, infertility, recurrent miscarriage, abdominal pain, and other complications later in pregnancy [1]. The true incidence of IUAs, however, is unclear since some patients are asymptomatic [2]. Clinical manifestations depend on the localization and the extent of endometrial injury within the uterine cavity [1]. Infection or trauma are the most common causes of such IUAs, especially after pregnancy. Up to 20–25% of patients treated with dilatation and curettage suffer from IUAs at 1–8 weeks postpartum [3].

Because the pathogenesis of IUAs has not been fully elucidated, the successful pregnancy rate remains low despite advances in therapeutic modalities [2]. However, treatment of IUAs aims to rebuild a normal uterine cavity and restore uterine function. The current therapeutic lines use hysteroscopic surgery for adhesion removal combined with hormonal therapy to stimulate endometrial regeneration from residual progenitor cells and endometrial stem cells [4]. Nevertheless, severe and dense IUAs represent a real therapeutic challenge and have a poor prognosis. Serious injury to the basal layer may lead to the loss of most of the endometrial cells and, eventually, the endometrium fails to regenerate. In serious injuries, treatment is difficult and has a poor prognosis [2].

Since Wood and Pena [5] proposed the use of estrogens to stimulate the regeneration of the endometrium and promote the re-epithelialization of the scarred surfaces, estrogen therapy has been commonly used as an adjuvant therapy after adhesiolysis since it improves endometrial regeneration and thus inhibits recurrent adhesions. Estrogen, while promising, often has a number of limitations such as a limited half-life in vivo, poor solubility in aqueous solutions, and low concentrations at the injured endometrium [6].

As a component of human endometrial tissues, endometrial stem cells have been recognized by their functional characteristics. These cells can regenerate the endometrium in many endometrial disorders. There are some definite markers of human endometrial stem cells that have been recognized such as CD146 and CD140b, which is known as platelet-derived growth factor receptor beta (PDGF-Rb) [7]. However, the regenerative process is disrupted in Asherman’s syndrome [8]. Mesenchymal stem cells (MSCs) have the ability to differentiate into different lineages of mesenchymal tissue [9]. Increasing evidence suggests that MSCs play a significant role in repairing injured tissues, not only by cellular differentiation but also via the secretion of a wide range of paracrine factors such as chemokines, growth factors, and cytokines into the environment. These paracrine factors, which have anti-inflammatory, antiscarring, and angiogenic effects, are the most biologically active components in the process of the MSC-induced reconstruction of injured tissues [10].

The therapeutic impact of applied MSCs largely relies on released factors, including extracellular vesicles (EVs) [11]. Recently, cell-derived EVs were demonstrated to be a unique mechanism of cell-to-cell communication [12]. EVs are defined as vesicles derived from the plasma membrane and discharged into the microenvironment by several cell types including stem cells and their progenitors [13]. EVs also transport microRNA (miRNA) and mRNA, which may induce epigenetic changes in target cells [14]. In addition, EVs may enhance an angiogenic program in endothelial cells or regulate the phenotypes of cells injured by a horizontal transfer with miRNA or mRNA [15].

Thus, in the present study, we evaluated the therapeutic potential effects of vesicles derived from umbilical cord MSCs (UCMSCs-EVs) alone or in combination with estrogen for treating an in-vivo rat model of IUA, utilizing the potential antifibrotic and anti-inflammatory properties of UCMSCs-EVs.

## Methods

### Experimental animals

Sexually mature and nulliparous female Albino rats (180–200 g), 6 weeks of age, were purchased from the Experimental Animal Unit, Faculty of Veterinary Medicine, Benha University, Egypt. The rats were bred and maintained in an air-conditioned animal house under specific pathogen-free conditions. All animals were housed in clean cages and given a standard diet and clean water ad libitum. The rats were subjected to a normal light/dark cycle (12-h light-dark cycle starting at 8:00 am) and room temperature (23 ± 3 °C) and allowed free access to chow and water. This study was carried out in strict accordance with the recommendations in the Guide for the Care and Use of Laboratory Animals of the National Institutes of Health (NIH publication No. 85–23, revised 1996). All protocols were approved by the institutional review board for animal experiments of the Faculty of Medicine, Benha University, Egypt.

### Preparation of human UCMSCs-EVs

#### Isolation of human umbilical cord MSCs

Human umbilical cord specimens were obtained in collaboration with the Labor and Delivery nursing staff using protocols approved by the ethical committee of the Faculty of Medicine, Benha University. After informed consent was obtained from the patient, four fresh cord samples were retrieved during cesarean deliveries from women with healthy pregnancies. Wharton’s jelly was harvested from term deliveries at the time of birth. The Wharton’s jelly was minced and incubated with the collagenase II enzyme (IgG, *Clostridium histolyticum*; Biological life science, USA) at 37 °C for 2 h. A strainer (Invitrogen, CA, USA) was used to remove tissue debris. Isolated cells were cultured and propagated in 10% fetal bovine serum (FBS) and 1× penicillin/streptomycin (Invitrogen, CA, USA) at 5% CO_2_ and 37 °C until reaching 70–80% confluence. After 1 week of culture, cells were washed with phosphate-buffered saline (PBS) and trypsinized with 0.25% trypsin for 5 min at 37 °C. After centrifugation, the cell pellets were resuspended and propagated in RPMI-160 medium as first-passage cultures [16].

#### Isolation of human UCMSCs- EVs

MSCs were obtained from supernatants of human cord mesenchymal stem cells (hUMSCs) as described above. The hUMSCs were then cultured in Dulbecco’s modified Eagle’s medium (DMEM) without FBS and with added 0.5% human serum albumin (HSA; Sigma-Aldrich, St. Louis, MO, USA) overnight. The viability of the cells cultured overnight was > 99% as detected by trypan blue exclusion. The conditioned medium was collected and stored at −80 °C, followed by centrifugation of the medium at 2000 g for 20 min to remove debris and then ultracentrifugation at 100,000 g in a SW41 swing rotor (Beckman Coulter, Fullerton, CA, USA) for 1 h at 4 °C. The EVs were washed once with serum-free M199 (Sigma-Aldrich) containing 25 mM HEPES (pH 7.4) and submitted to a second ultracentrifugation under the same conditions. The EVs were stored at −80 °C for experiments [17]. The protein content of the isolated EVs was assessed by a Bradford protein assay kit. The dose of injected EVs was adjusted to 100 μg protein/suspended in 0.2 ml PBS [18].

#### Characterization of hUCMSCs-EVs

EVs were fixed with 2.5% glutaraldehyde in HSA for 2 h. After they were washed, the EVs were ultracentrifuged and suspended in 100 μl HSA. A total of 20 μl of EVs was loaded onto a formvar/carbon-coated grid, negatively stained with 3% aqueous phosphor-tungstic acid for 1 min, and observed by transmission electron microscopy (TEM; HITACHI, H-7650, Japan) [19]. Additionally, PKH26 (Sigma-Aldrich, St. Louis, MO, USA) was performed to confirm the exosome localization within the uterine tissue.

### Induction of intrauterine adhesions

Rats were used to construct the experimental model. Under ketamine (50 mg/kg, intraperitoneally) anesthesia, the abdomen was shaved and prepared with an iodophors solution. The uterine horns were exposed by an abdominal midline incision, and a piece of gauze was used to wrap the right uterine horn to prevent abdominal viscera. To set up the experimental model, we injected 0.1 ml trichloroacetic acid (Sigma Chemical Co., St. Louis, MO) into the right uterine horn. The left horn was used as a control [16].

### Experimental design and treatment protocol

Forty-nine adult female albino rats were cycle synchronized according to a vaginal smear analysis which allows for the determination of the phases of the 4-day estrus cycle: proestrus, estrus, metestrus, and diestrus.

The animals were divided into five groups.

#### Group I (control group)

Twenty-one rats were fed a regular chow diet for 8 weeks. The rats were subdivided equally into three subgroups (seven animals per group): subgroup a, rats with no intervention; subgroup b, sham group; and subgroup c, same as the sham group, but after 2 weeks a relaparotomy was performed, and the rats were injected with 0.2 ml normal saline (as a vehicle for hUCMSCs-EVs) in the right uterine horn followed by three consecutive intraperitoneal injections of normal saline at 5-day intervals.

#### Group II (IUAs group)

Seven rats underwent induction of intrauterine adhesions and were then left without any intervention or treatment.

#### Group III (IUAs + estrogen group)

Seven rats underwent induction of intrauterine adhesions, followed by daily oral estrogen (estradiol oral 2 mg tablet; Reliable Canadian Pharmacy, Mississauga, Ontario, Canada) (0.1 mg/kg) [20] initiated in the second week of the experiment.

#### Group IV (IUAs + hUCMSCs-EVs group)

Seven rats underwent induction of intrauterine adhesions. At the end of the second week, a relaparotomy was performed, and hUCMSCs-EVs (100 μg/kg/dose suspended in 0.2 ml normal saline) [18] were injected into the right uterine horn followed by three consecutive intraperitoneal injections of hUCMSCs-EVs at 5-day intervals.

#### Group V (IUAs + estrogen + hUCMSCs-EVs)

Seven rats underwent induction of intrauterine adhesions. At the end of the second week, a relaparotomy was performed, and hUCMSCs-EVs (100 μg/kg/dose suspended in 0.2 ml normal saline) were injected into the right uterine horn followed by three consecutive intraperitoneal injections of hUCMSCs-EVs at 5-day intervals combined with daily 0.1 mg/kg oral estrogen.

### Sampling

After 8 weeks, the rats were sacrificed, and the bilateral uterine horns were resected. The specimens were evaluated by light microscopy with hematoxylin and eosin (H&E) and Masson’s trichrome staining. An immunohistochemical evaluation for transforming growth factor (TGF)-β and vascular endothelial growth factor (VEGF) was also performed. Fresh endometrial tissue specimens were kept frozen at −80 °C for later quantitative real-time polymerase chain reaction (qRT-PCR) to assess the gene expression of interleukin (IL)-1, IL-6, tumor necrosis factor (TNF)-α, CD140b (an endometrial stem cell marker), and RUNX2, while a Western blot was performed for collagen I and β-actin.

### Histopathological analysis

The uterine tissues were excised, and the sections were fixed in 10% buffered formol saline, processed as 4–6 μm thick paraffin sections, and then mounted on glass slides for H&E and Masson’s trichrome staining [21].

### Immunohistochemistry analysis

Immunohistochemistry staining was performed to detect TGF-β (a fibrotic marker) and VEGF (a vascular marker). The anti-TGF-β antibodies were obtained from Lab Vision Corp., Neo-markers, Inc./Lab Vision, Fremont, California, USA, while the anti-VEGF antibodies were obtained from ab51745 rabbit polyclonal antibodies [22].

### Morphometric study

The mean area percentage of collagen fiber deposition as indicated by Masson’s trichrome and the mean area percentage of TGF-β and VEGF expression were quantified for five images from five nonoverlapping fields from each rat of each group using the Image-Pro Plus program version 6.0 (Media Cybernetics Inc., Bethesda, Maryland, USA).

### Determination of the gene expression of IL-1, IL-6, TNF-α, CD104b, and RUNX2

#### Total RNA extraction and reverse transcription

Total RNA was extracted from tissue samples using a RNeasy mini kit (Qiagen, Germany) according to the manufacturer’s instructions. For cDNA synthesis, extracted RNA samples were quantified using a NanoDrop One spectrophotometer (Thermo Fisher Scientific, USA) and (1 μg) reverse transcribed with a T100 Thermal Cycler (Bio-Rad, USA) using a QuantiTect Reverse Transcription Kit (Qiagen, Germany), following the manufacturer’s instructions [23].

#### Quantitative real-time PCR

Real-time PCR was performed using a QuantiTect SYBR Green PCR Kit (Qiagen, Germany) on a StepOnePlus Real-Time PCR System (Life Technologies, USA). The sequences of the specific primers used were as follows: IL-1: 5′-GCAATGGTCGGGACATAGTT-3′ (forward), 5′-AGACCTGACTTGGCAGAGGA-3′ (reverse); IL-6: 5′-TCTCTCCGCAAGAGACTTCCA-3′ (forward), 5′-ATACTGGTCTGTTGTGGGTGG-3′ (reverse); TNF-α: 5′-ACCACGCTCTTCTGTCTACTG-3′ (forward), 5′-CTTGGTGGTTTGCTACGAC-3′ (reverse), [24]; CD140b: 5′- CACCATTTCGAGCACCTTTGT -3′ (forward), 5′- AGGGCACTCCGAAGAGGTAA-3′ (reverse) [25]; RUNX2: 5′- GCCCAGGCGTATTTCAGATG-3′ (forward), 5′-GGTAAAGGTGGCTGGGTAGT-3′ (reverse) [26]; and GAPDH: 5′- ACCACAGTCCATGCCATCAC -3′ (forward), 5′-TCCACCACCCTGTTGCTGTA-3′ (reverse) [27]. The mRNA expression of each sample was determined after correction by *GAPDH* expression. The relative expression was calculated using the 2^–ΔΔCT^ method. The results were expressed as the *n*-fold difference relative to the control group. Each sample was assayed three times.

### Western blot

The collagen type І and β-actin antibodies were purchased from Thermo Scientific (MA1–26771). Protein was extracted from uterine tissue using RIPA lysis buffer which was obtained from Bio BASIC Inc. (Marhham, Ontario, Canada). The extracted protein was separated by SDS-PAGE on 4–20% polyacrylamide gradient gels. After incubation in 5% nonfat dry milk, Tris-HCL, and 0.1% Tween 20 for 1 h, collagen-I monoclonal antibody and β-actin antibody were added to one membrane containing specimen samples and incubated at 4 °C overnight. Appropriate secondary antibodies were incubated for 2 h at room temperature. After washing twice with 1× TBST, a densitometric analysis of the immunoblots was performed to quantify the amounts of collagen I and β-actin against the control sample by total protein normalization using Image analysis software on the Chemi Doc MP imaging system (Version 3) produced by Bio-Rad (Hercules, CA).

### Statistical analysis

Statistical analysis was performed using the statistical software package SPSS for Windows (Version 16.0; SPSS Inc., Chicago, IL, USA). Differences between groups were evaluated using a one-way analysis of variance (ANOVA) with a post-hoc test (LSD). For each test, all the data are expressed as the mean ± standard deviation (SD), and a *P* value < 0.05 was considered significant.

## Results

### Exosomes characterization

**Fig. 1:**
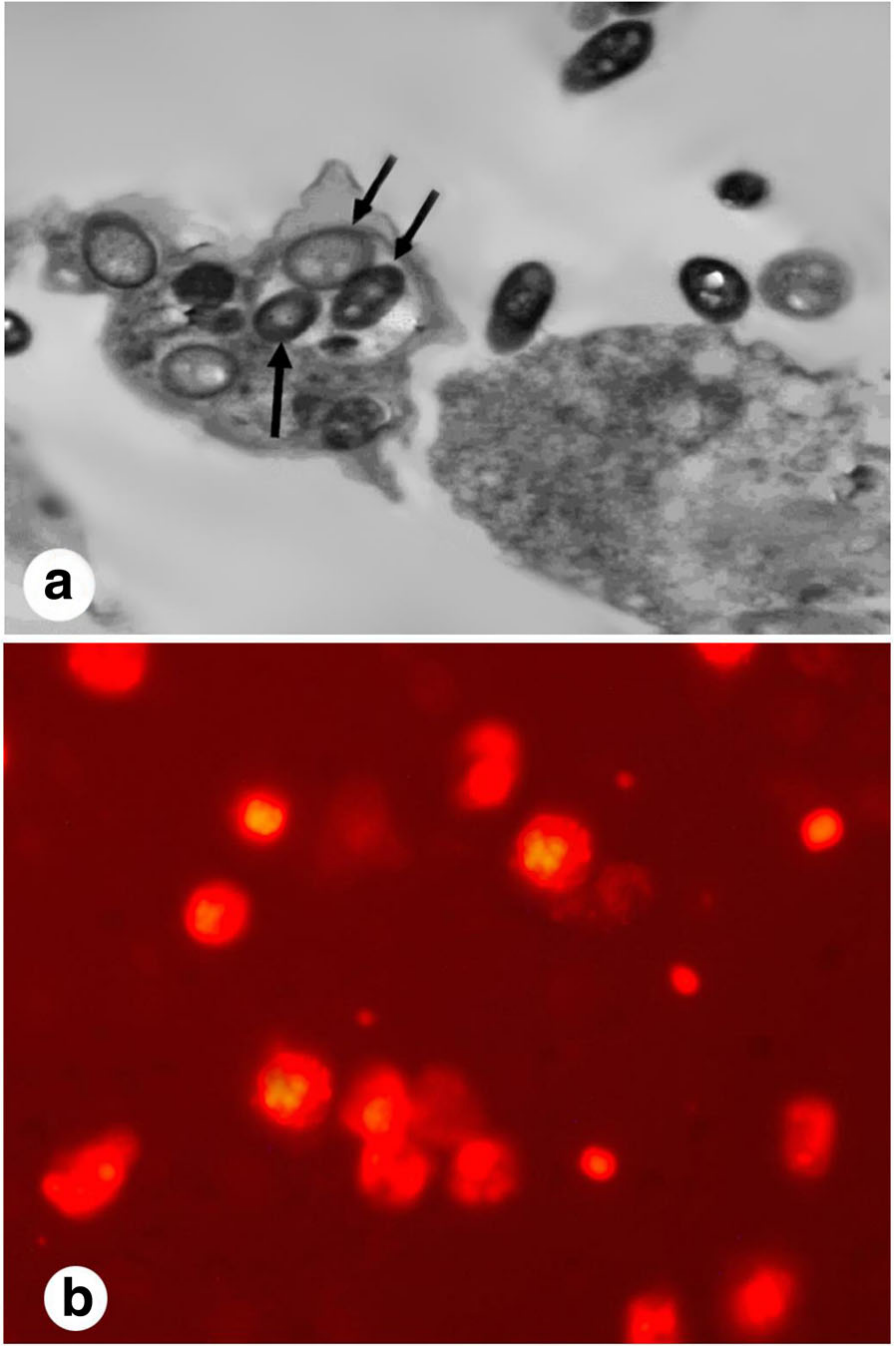
Transmission electron microscopy (TEM) of exosomes showing a spheroid double membrane bound morphology (arrows) with a diameter of 40–100 nm (**a**). Additionally, the exosomes were detected in uterine tissues by PKH26 (**b**)

A transmission electron microscopic examination of purified exosomes demonstrated their characteristic spheroid double membrane-bound morphology and indicated a diameter of 40–100 nm (Fig. 1a). Additionally, exosomes in uterine tissue were detected by PKH26 (Fig. 1b).

### Histological results

#### H&E results

**Fig. 2:**
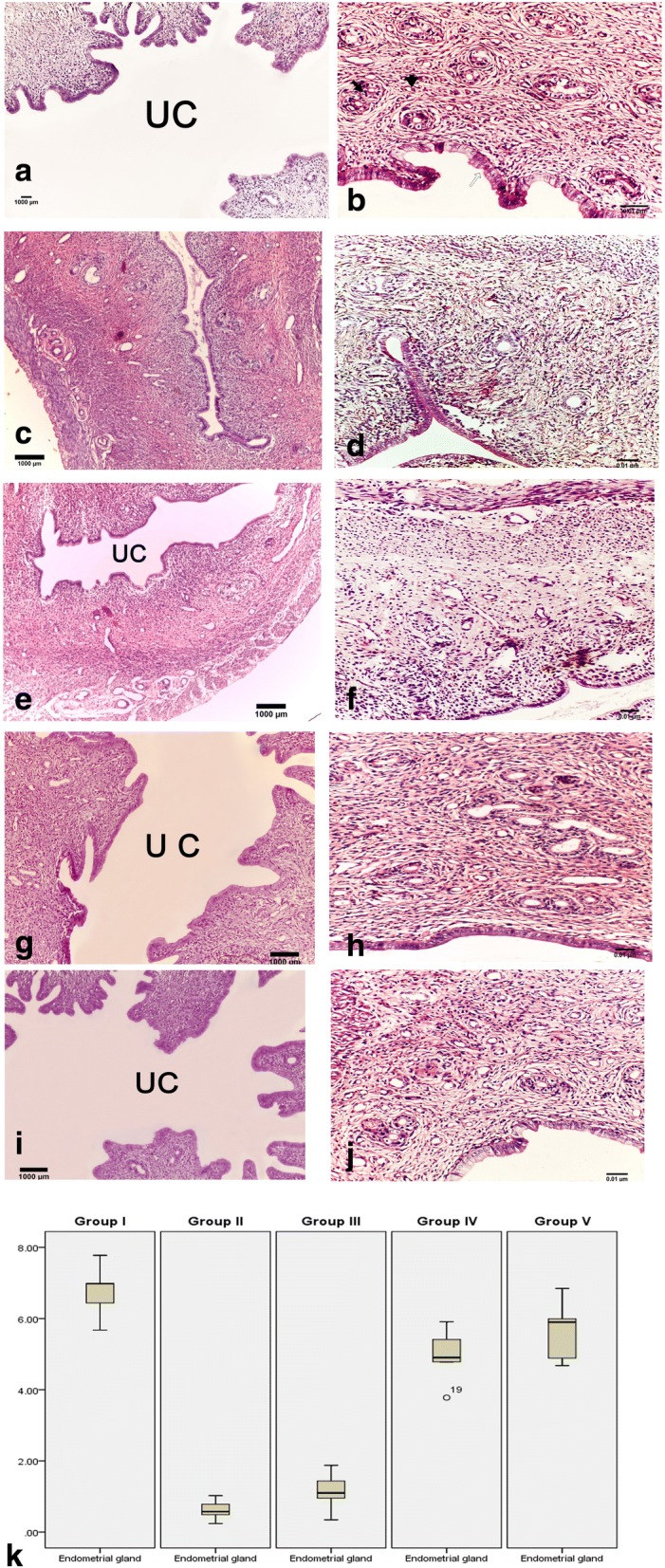
The H&E-stained uterine sections revealed that hUCMSCs-EVs alleviate the inflammatory response in an experimentally induced IUA model in rats. In the control group, the endometrial surface is lined by high columnar epithelial cells (ECs) and round or oval uterine glands (UGs) in the submucosa and basal layer (**a**). The uterine cavity (UC) is widely opened (**b**). After 30 days of induction of IUAs, the surface in group II rats (IUAs group) was covered by flat and low columnar epithelial cells with few glands under the epithelial layer (**c**) and narrowing of the UC (**d**). In group III, the endometrial surface is lined by low columnar ECs and few numbers of UGs (**e**). The UC of group III is narrow (**f**). In group IV, the endometrial surface is covered by columnar ECs and an increased number of UGs (**g**). The UC of group IV is wide (**h**). In group V, the endometrial surface is lined by high columnar ECs and numerous UGs (**i**). The UC of group V is wide (**j**). Box blot of the mean number of endometrial glands in all groups (**k**); data are shown as mean ± SD, *n* = 5

An examination of the H&E-stained uterine sections revealed that the endometrium contained surface columnar epithelium cells overlying a thick layer of lamina propria with compact stromal cells, numerous tiny blood vessels (BV), and endometrial glands (EG). In the control group (group I), the endometrial surface was lined with simple high columnar epithelial cells (ECs). Round or oval glands were primarily found in the submucosa and basal layer, and there were large openings at the endometrial surface (Fig. 2a). The uterine cavity (UC) was widely open (Fig. 2b). Thirty days after IUA induction, the uterine surface in group II (IUAs group) was covered by flat and low columnar epithelial cells with a few glands under the epithelial layer (Fig. 2c). Additionally, there was narrowing in the uterine cavity with intrauterine adhesions (Fig. 2d). In group III (IUAs + estrogen group), low columnar endometrial epithelial cells were seen with few glands and a narrow uterine cavity (Fig. 2e, f). However, in groups IV (IUAs + hUCMSCs-EVs) and V (IUAs + estrogen + hUCMSCs-EVs), the endometrial surface epithelial cells were high columnar cells with a greater number of glands and a wider uterine cavity. These results were more pronounced in group V (Fig. 2i, j) than in group IV (Fig. 2g, h).

#### Masson’s trichrome stain

**Fig. 3:**
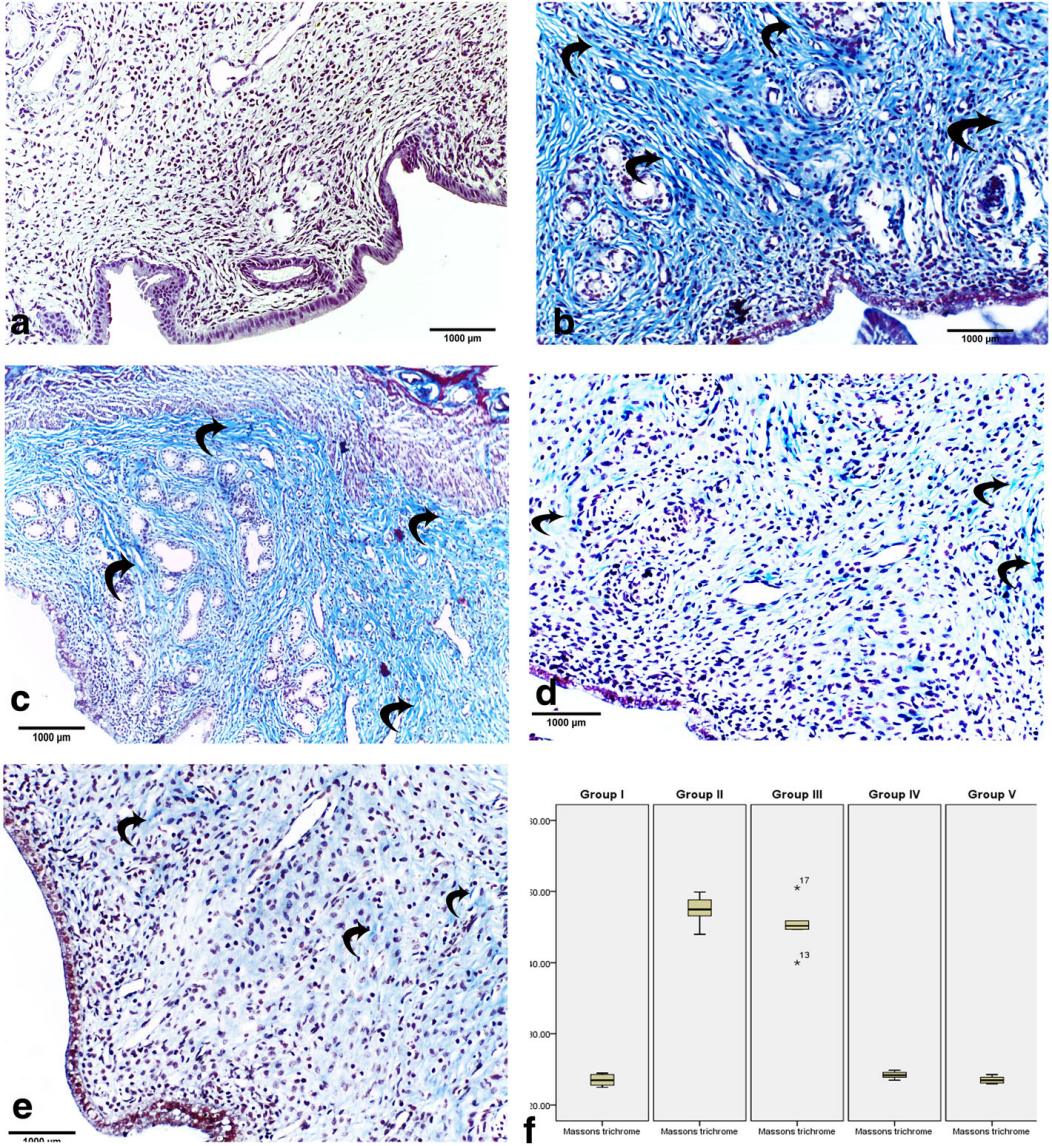
Masson’s trichrome staining of uterine sections was performed to assess the extension of fibrosis in an injured endometrium in an IUA model with/without treatment. Stained tissue sections revealed that the endometrial stroma in group I (control) had almost no collagen fiber deposition (**a**). However, groups II (IUAs) and III (IUAs + estrogen) showed a marked increase in collagen fiber deposition (**b**, **c**). Groups IV (IUAs + hUCMSCs-EVs) and V (IUAs + estrogen + hUCMSCs-EVs) showed a decrease in the amount of collagen fiber deposition in the endometrium (**d**, **e**). Box blot showing the mean area percentage of collagen fibers in all groups (**f**). Data are expressed as the mean ± SD, *n* = 5. Curved arrows indicate the collagen fiber deposition

To assess the extent of fibrosis in the injured endometrium in an IUA model, we performed Masson trichrome staining of collagen fibers to detect endometrial fibrosis. In group I (control), the endometrial stroma had almost no collagen fiber deposition (Fig. 3a). However, in group II (IUAs group) and group III (IUAs + estrogen), there were marked increases in the collagen fiber deposition compared with that in the control group (Fig. 3b, c). Group IV (IUAs + hUCMSCs-EVs) and group V (IUAs + estrogen + hUCMSCs-EVs) revealed a marked decrease in the amount of collagen fiber deposition in the endometrium compared with that in group II (Fig. 3d, e).

#### Immunohistochemistry results

**Fig. 4:**
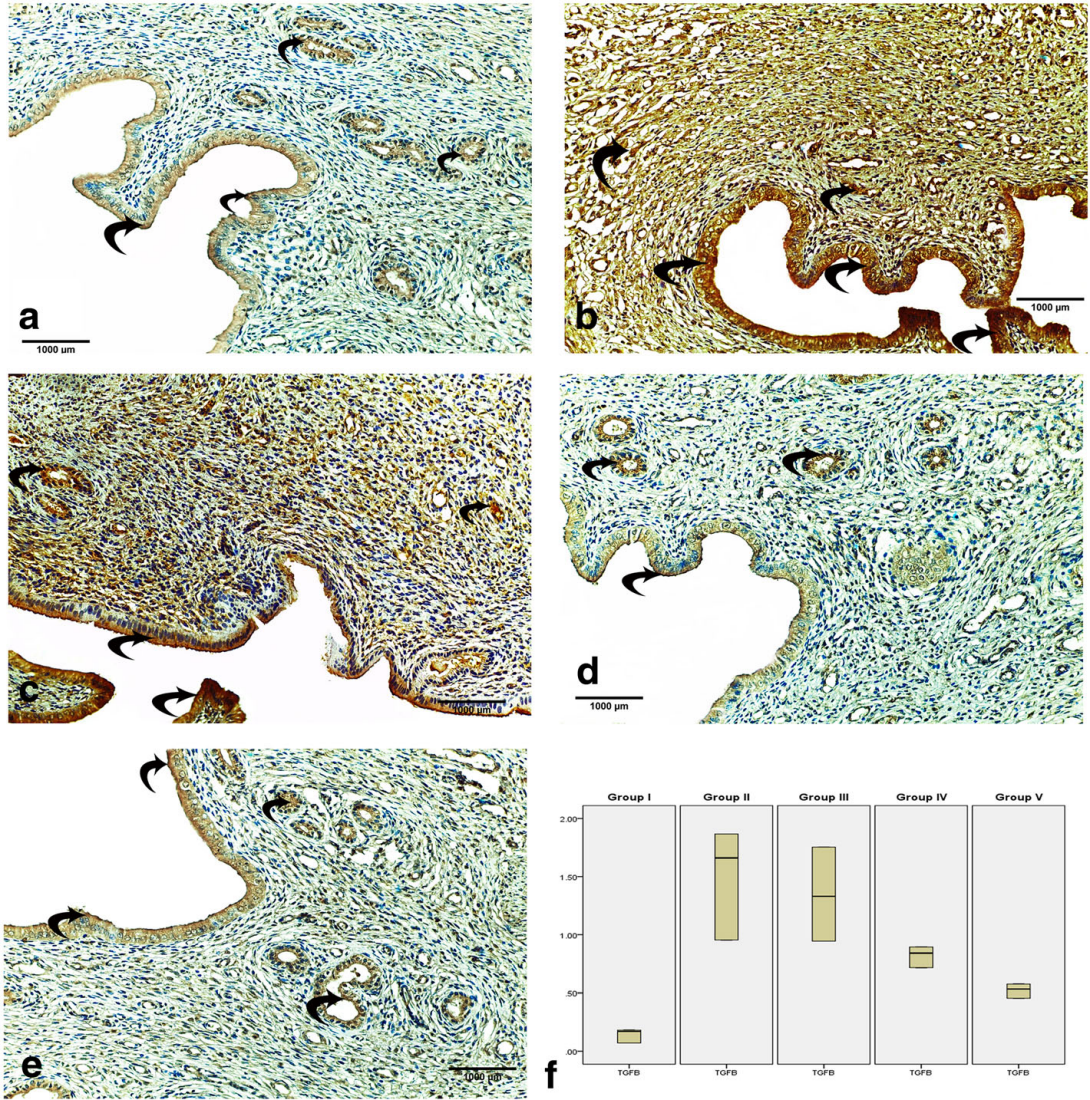
The transforming growth factor (TGF)-β immunoexpression in the endometrium. In group I (control), the reaction is weak in endometrium (**a**). In group II (IUAs), the reaction is very strong in the endometrium (**b**). In group III (IUAs + estrogen), the reaction is mildly lower than that of group II (**c**). In group IV (IUAs + hUCMSCs-EVs), the reaction is weak in the endometrium (**d**). In group V (IUAs + estrogen + hUCMSCs-EVs), the reaction is very weak in the endometrium (**e**). Box blot of mean area percentage of TGF-β immunoexpression in all groups (**f**). Data are shown as mean ± SD, *n* = 5. Curved arrows indicate TGF-β-positive cells

To assess the extent of IUA, we evaluated TGF-β immunoexpression since TGF-β plays an important role in fibrosis formation. TGF-β was mainly expressed in the nucleus and in the cytoplasm of stromal and epithelial cells. TGF-β immunoexpression in the endometrium of group I (control group) was very weak (Fig. 4a). The immunoexpression of TGF-β in group II (IUAs group) was significantly higher than that in the control group (Fig. 4b). TGF-β immunoexpression in group III (IUAs + estrogen) was slightly lower than that in the IUAs group (Fig. 4c). However, TGF-β immunoexpression in groups IV and V was weak compared with that in group II (Fig. 4d, e).

**Fig. 5:**
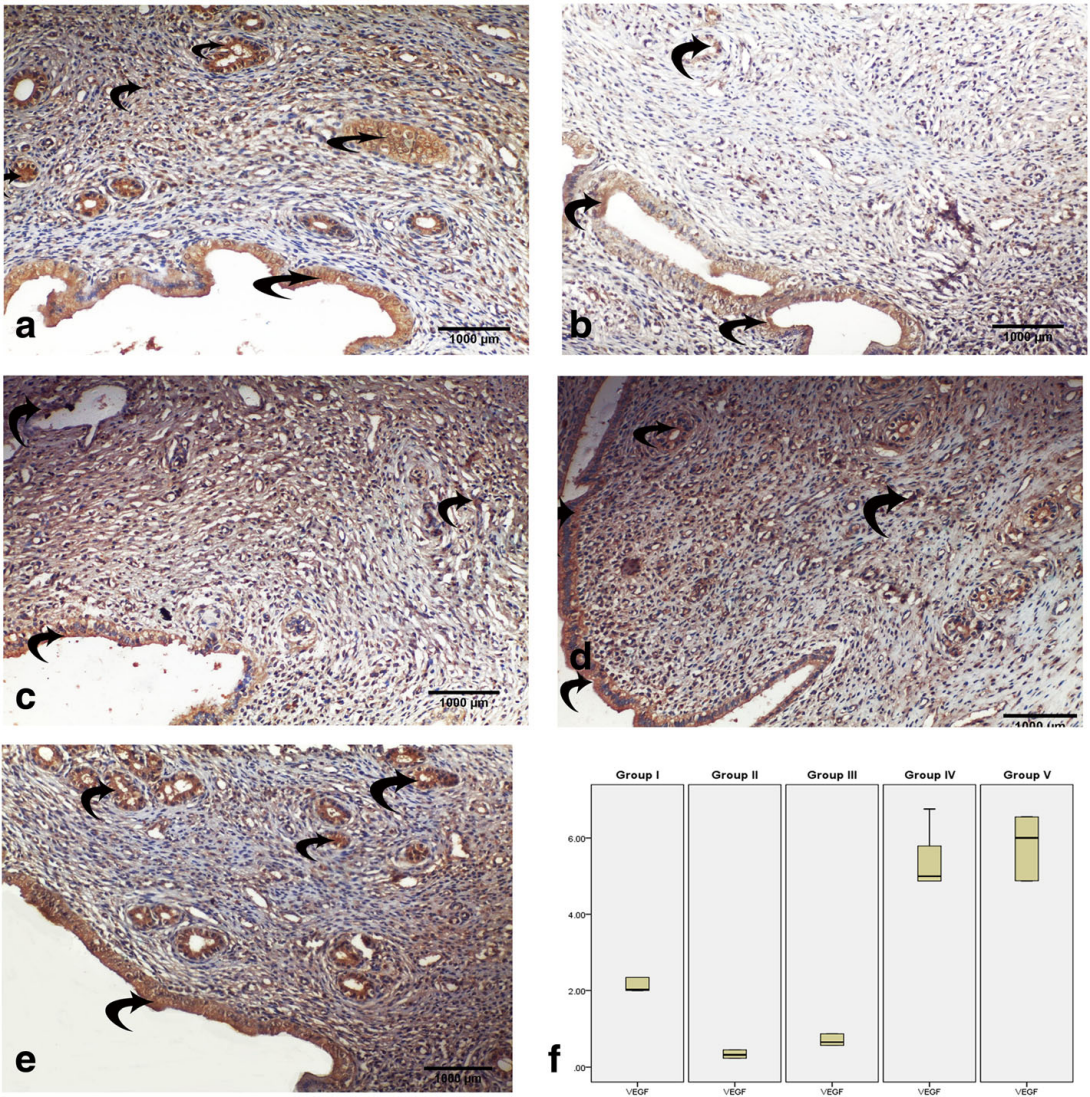
The vascular endothelial growth factor (VEGF) immunoexpression in the endometrium. In group I (control), the reaction is strong in the endometrium (**a**). In group II (IUAs), the reaction is very weak in the endometrium (**b**). In group III (IUAs + estrogen), the reaction is weak in the endometrium (**c**). In group IV (IUAs + hUCMSCs-EVs), the reaction is strong in the endometrium (**d**). In group V (IUAs + estrogen + hUCMSCs-EVs), the reaction is very strong in the endometrium (**e**). Box blot of mean area percentage of VEGF immunoexpression in all groups (**f**). Data are shown as mean ± SD, *n* = 5. Curved arrows indicate VEGF-positive cells

To assess endometrial vascularity, we evaluated VEGF immunoexpression in all groups. VEGF immunoexpression in the endometrium of group I (control group) was strong (Fig. 5a), whereas the immunoexpression of VEGF in group II (IUAs group) was weak compared with that in the control group (Fig. 5b). VEGF immunoexpression in group III (IUAs + estrogen) was slightly greater than that in the IUAs group (Fig. 5c). In contrast, VEGF immunoexpression in groups IV and V was strong compared with that in group II (Fig. 5d, e). Cytoplasmic expression was seen in all groups.

#### Morphometric and statistical results

##### Mean number of endometrial glands in the uterine sections

The mean number of endometrial glands in control group I was 6.77 ± 0.78. Marked depletion of the endometrial gland count was found in group II (IUAs group) and group III (IUAs + estrogen), totaling 1.14 ± 0.37 and 1.69 ± 0.57, respectively, and represented a significant decrease compared with that in the control group. A statistically significant increase in the number of endometrial glands was found in groups IV and V, with 4.96 ± 0.80 and 5.66 ± 0.88 glands, respectively, compared with that in group II (Fig. 2k).

##### Mean area percentage of collagen fiber deposition in the endometrium

The mean area percentage of collagen fiber deposition is presented in Fig. 3f. In group II (IUAs group), there was a significant increase in the area percentage of collagen fiber deposition compared with that in the control group. In group III (IUAs + estrogen), a nonsignificant difference was seen in endometrial fibrosis between the IUAs group (group II) and group III (IUAs + estrogen), with the latter group having significantly greater endometrial fibrosis than the control group. In group IV (IUAs + hUCMSCs-EVs), the area percentage of collagen deposition was significantly decreased compared with that in group II (IUAs group). In group V (IUAs + estrogen + hUCMSCs-EVs), the area percentage of collagen deposition was significantly reduced compared with that in group II and was nearly similar to that in the control group. Broadly, the changes in endometrial fibrosis were inversely related to the number of glands in the rats in each group.

##### Mean area percentage of the TGF-β and VEGF immunoreactivity in the endometrium in all groups

The mean area percentage of TGF-β is shown in Fig. 4f. In group II (IUAs group), there was a significant increase in the area percentage of TGF-β immunoexpression compared with that in the control group. Nonsignificant differences were noticed between groups II and III. However, a significant decrease occurred in groups IV and V compared with that in group II (IUAs group).

Similarly, the mean area percentage of VEGF is shown in Fig. 5f. In group II (IUAs group), there was a significant decrease in the area percentage of VEGF immunoexpression compared with that in the control group. Nonsignificant differences were noticed between groups II and III. However, the mean area percentage of VEGF in groups IV and V was significantly higher than that in group II (IUAs group).

### Gene expression results of *IL-1*, *IL-6*, *TNF-α*, *CD140b*, and *RUNX2* genes in all experimental groups

**Fig. 6:**
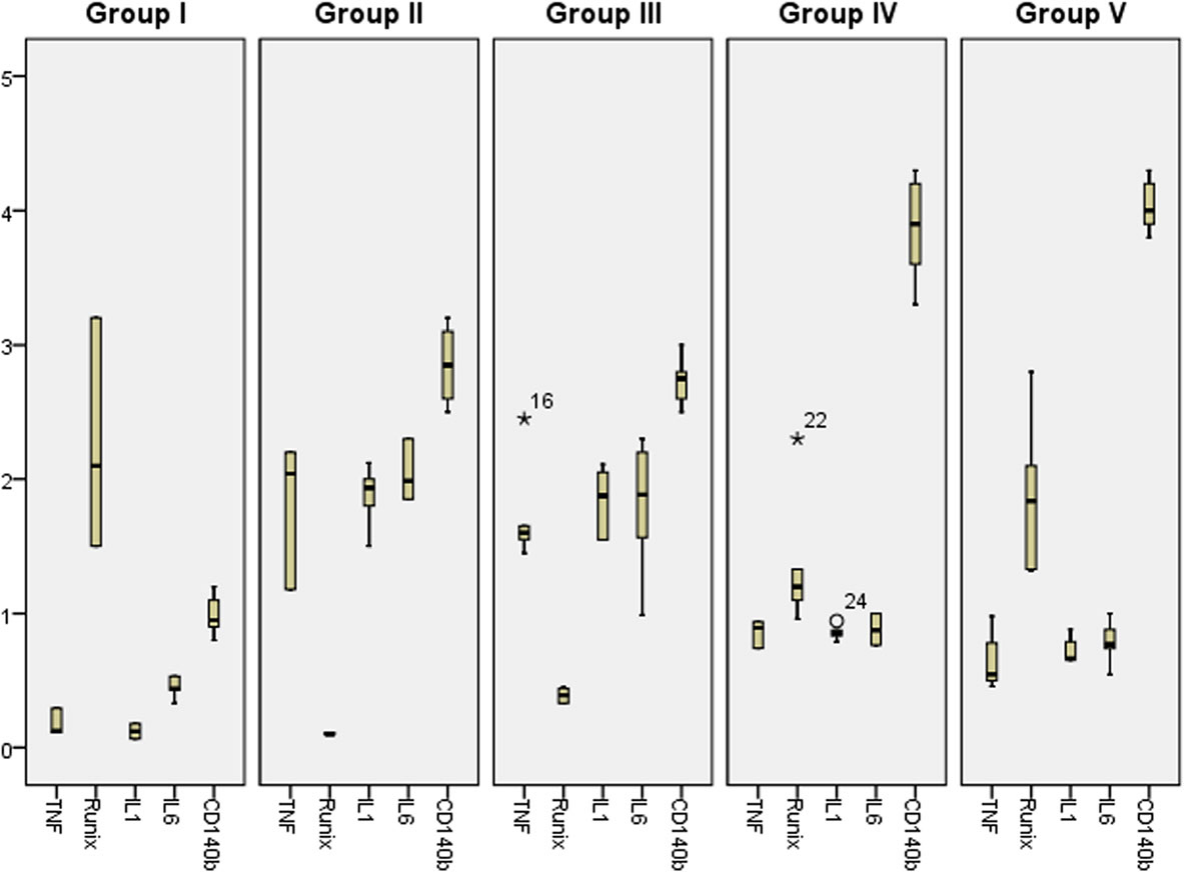
Quantitative analysis for relative interleukin (IL)-1, IL-6, tumor necrosis factor (TNF)-α, CD140b, and RUNX2 gene expression after the indicated treatment of IUAs with estrogen and/or hUCMSCs-EVs. Data are shown as mean ± SD, *n* = 5

Gene expression of inflammatory cytokines (*IL-1*, *IL-6*, and *TNF-α*) was significantly downregulated in groups IV and V when compared with the expression in group II (IUAs). However, CD140b, a marker of endometrial stem cells, showed significant upregulation in groups II and III when compared with the expression in group I. In addition, CD140b was significantly upregulated in groups IV and V compared with the expression in group II. RUNX2 (an antifibrotic factor) expression was significantly increased in groups IV and V compared with that in group II (Fig. 6).

Notably, estrogen treatment (group III) produced nonsignificant effects compared with IUA (group II) on both inflammatory cytokine and fibrotic factor expression. Thus, treatment with estrogen alone was not a sufficiently effective therapy.

### Collagen I and β-actin detection by Western blotting

**Fig. 7:**
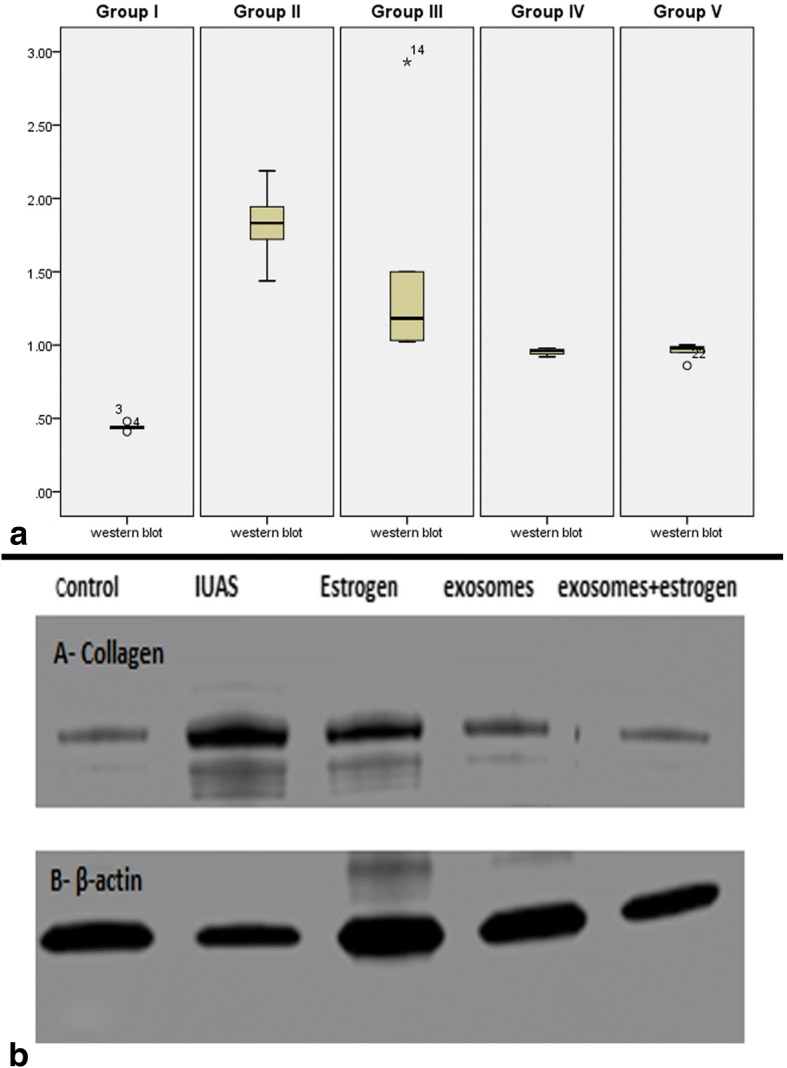
Western blot assay and downstream target proteins for collagen-I and β-actin expression after treatment with estrogen and/or hUCMSCs-MVs (**b**) and quantified using Image analysis software on the Chemi Doc MP imaging system. Data are shown as mean ± SD, *n* = 5 (**a**)

Collagen I and β-actin protein expression was higher in group II (IUAs) and group III (IUAs + estrogen) than in group I (control), while expression was lower in groups IV and V (Fig. 7). Collagen expression was normalized relative to the expression of β-actin.

## Discussion

Transplantation of hUCMSCs has been considered to have the potential for therapeutic effects on tissue regeneration and organ repair in the treatment of certain inflammatory disorders and destructive diseases [28, 29]. Interestingly, MSCs have been found to secrete exosomes or vesicles, which are enriched in the extracellular environment. Vesicles have been considered as vital mediators of cellular communication and may regulate various physiological and pathological processes by transferring membrane proteins, mRNAs, and miRNAs to recipient cells [30, 31]. Recent studies have demonstrated that vesicles can act during different stages of the inflammatory response by transporting bioactive factors, and MSC exosomes have been reported to suppress inflammation in different animal models [32]. These cells seem to be working intelligently and systematically to restore the physiological status quo [29].

The incidence of Asherman’s syndrome or intrauterine adhesions (IUAs) has been increasing over the last few decades due to an increasing number of cesarean sections in addition to increased awareness and more advanced diagnostic tools. Hence, there has been an increasing demand for the development of new approaches for the treatment of Asherman’s syndrome. IUAs prevent the endometrium from growing and lead to infertility. Various treatment regimens including high doses of estrogen and progesterone have been tried for IUAs; however, none of them has proven effective, which is a problem that has spurred investigators to continue searching for new treatment options.

In this study, we mainly focused on the therapeutic effects of human umbilical cord mesenchymal stem cells (hUCMSCs) as a source for extracellular vesicles, which are a major part of the paracrine mechanism of MSCs, for the treatment of IUAs induced in rats. Notably, we used two routes for delivering hUCMSCs-EVs into the uterine tissues in our study. Intrauterine application of hUCMSCs-EVs was primarily employed, but the rest of the applications were intraperitoneal to imitate the intravenous route. Intrauterine application was used because it improved neutrophil activation and the C-reactive protein-associated injurious effects on migration, differentiation, and survival of cells during the acute phase of injury. The intraperitoneal route has systemic anti-inflammatory effects on the circulating lymphocytes as manifested by the expansion of IL-6 and TGF-β1 levels [33]. However, the intrauterine route may be a better means to provide an efficient number of hUCMSCs-EVs to the endometrial cells to encourage the horizontal transmission of the hUCMSCs-EV mRNA and miRNA cargo to the endometrial cells. Thus, systemic hUCMSCs-EV administration may regulate circulating immune cells via their anti-inflammatory effects, while intrauterine EV administration may control the local environment and trigger endogenous progenitor cells to potentiate the concession of endometrial cells to the effects of the hUCMSCs-EVs cargo, thereby encouraging cell recovery [34].

In this study, we found that group II rats (IUAs), unlike control rats, had impaired endometrial epithelial cells, a lower number of endometrial glands, higher inflammatory cell infiltration, poor vascularity, and a severe narrowing of the uterine cavity with dense endometrial fibrosis as confirmed by H&E and Masson’s trichrome staining. Notably, massive collagen fiber deposition was found in group III (IUAs + estrogen), and no glandular proliferation was indicated by H&E and Masson’s trichrome staining (Figs. 2 and 3). These results agreed with the results of Sabry et al. [34], who revealed dense fibrosis, severe inflammation, a necrotic endothelium, mild vascular proliferation, and no glandular proliferation in a rat model for uterine fibrosis treated only with estrogen. Interestingly, the current results from group IV (IUAs + hUCMSCs-EVs) demonstrated the presence of mild fibrosis with a patent uterine cavity and strong glandular and vascular proliferation. These findings are in accordance with Alawadhi et al. [35], who saw no significant difference in fibrosis between the normal control group and a bone marrow-derived mesenchymal stem cell-transplanted group based on a histological examination. A more recent study reported that treatment of induced IUAs with stem cells alone restored the patency of the uterine cavity, reduced inflammation and fibrosis, and enhanced vascular and glandular proliferation [34]. The results of the present study revealed that the best results were obtained in group V (IUAs + estrogen + hUCMSCs-EVs); there was very minimal or even no fibrosis with an obvious patent uterine cavity, and strong vascular and glandular proliferation. Our results support recent reports of a patent uterine cavity, the absence of fibrosis and inflammation with intense vascular proliferation and intense glandular proliferation in induced IUA treated with stem cells [34]. These histological changes were coincidently associated with the high immunoexpression of TGF-β and VEGF in group II, indicating massive uterine fibrosis and diminished uterine vascularization in an IUA rat model.

To better capture the global changes, we used immunohistochemical analysis to quantify cell number in addition to profiling marker expression. We found significant upregulation of the gene expression of IL-1, IL-6, and TNF-α, and significant downregulation of RUNX2 in IUAs (group II) compared with the expression in the control (group I). Some fibrotic markers such as connective tissue growth factor (CTGF), TGF, and collagen I, and some inflammatory cytokines such as IL-1 and IL-6 are well known to be upregulated in endometriosis models [36]. Moreover, IL-1 is an important mediator of the inflammatory response and is involved in a variety of cellular activities, including cell proliferation, differentiation, and apoptosis [37]. Our data are in accordance with a concept proposed by Liu [38], who revealed that IL-1 contributes to the development of fibrosis through various mechanisms such as the regulation of myofibroblast activation, the induction and secretion of chemokines (e.g., IL-8 and matrix metalloproteinase (MMP)), and the turnover of the extracellular matrix. Furthermore, IL-1 in combination with TNF-α and interferon (IFN)-γ is able to increase the TGF-β-induced epithelial-mesenchymal transition (EMT), which is an important cellular process during fibrogenesis. On the other hand, IL-6 is a proinflammatory cytokine [39] that contributes to development of fibrosis by modulating TGF-β signaling and stimulating the proliferation of fibroblasts, as well as collagen production. TGF-β has an immune-modulatory effect through inhibiting the proliferation of effector T cells and augmenting the proliferation of the regulatory CD4 CD25 FOXP3 T-cell population [40]. This immune-modulatory effect leads to differentiation and sometimes to dedifferentiation and/or the rearrangement of stromal cells [41].

Furthermore, group II showed a significant increase in TGF-β expression in the endometrium. TGF-β is an important cytokine for the induction of the EMT during fibrosis. TGF-β signaling induces the EMT through various signaling mechanisms and is the predominant agent mediating these fibrotic changes. Chronic exposure to TGF-β induces the transition of normal cells to collagen-producing mesenchymal cells, thus increasing the expression of collagen and inducing a cytoskeletal rearrangement that resembles the EMT [42]. This effect was confirmed in the present study by the evaluation of the gene expression of CD140b (an endometrial stem cell marker), which revealed that CD140b was upregulated in the endometrial tissue of group II (IUAs group) compared with the expression in the control group (group I). The endometrial stem cells and microenvironment (niche) are responsible for regenerating the endometrium. Therefore, the increased expression of fibrosis markers including TGF-β and collagen (Figs. 4 and 7) indicates an altered endometrial stem cell differentiation niche. As a result, the endometrial stem cells were incorrectly induced to become fibroblasts, leading to IUAs. In agreement with these findings, Hu et al. [7] showed that the endometrial stem cell markers CD146 and CD140b and the fibrotic markers TGF-β, CTGF, collagen I, and collagen III were abnormally expressed in the endometrium in an intrauterine adhesions model in female rats, indicating endometrial stem cells were wrongly induced to become fibroblasts, resulting in IUAs.

In the present study, in group III, estrogen administration alone significantly upregulated the inflammatory cytokines IL-1, IL-6, and TNF-α and induced a moderate increase in the gene expression of CD140b and the fibrotic markers collagen I and β-actin with an associated significant increase in the immunoexpression of TGF-β compared with the expression in the control group (group I). Because estrogen and some growth factors regulate endometrial stem/progenitor cells, they may affect the microenvironment of the endometrial stem cells. Estrogen causes changes in ovarian steroids, leading to alterations in the endogenous hormonal environment, with the interruption of the endometrial environment necessary for effective embryo implantation due to the clear rise in TGF-β expression [43]. Rageh et al. [44] showed that CTGF and TGF-β mRNA were powerfully stimulated after estradiol administration in ovariectomized mice. Moreover, collagen biosynthesis was increased by small doses of estradiol in cultured leiomyoma cells [45]. Several previous studies have demonstrated that estrogen can enhance the development of endometriotic lesions by increasing TNF-α levels through mast cell activation [40]. In contrast, Westphal and colleagues [46] showed that 17β-estradiol reduced the development of myocardial fibrosis by reducing the gene expression of CTGF and TGF-β.

Interestingly, the current results from group IV (IUAs + hUCMSCs-EVs) demonstrated the presence of mild fibrosis with a patent uterine cavity and strong glandular and vascular proliferation. These findings are concordant with those in a previously published work by Alawadhi et al. [35] who demonstrated nonsignificant differences in fibrosis based on a histological examination between the normal control group and a bone marrow-derived mesenchymal stem cell-transplanted group. A more recent study reported that treatment of induced IUAs with stem cells alone restored the patency of the uterine cavity, reduced inflammation and fibrosis, and enhanced vascular and glandular proliferation [34].

Additionally, our findings showed a significant reduction in collagen fiber deposition, confirmed by Western blot, in group IV (IUAs + hUCMSCs-EVs) compared with that in untreated rats in the IUAs group (group II) and those treated with estrogen alone (group III) (Fig. 7). This finding may be explained by the antifibrotic and anti-inflammatory impact of the hUCMSCs-EVs. In line with these results, Xu et al. [47] have recently demonstrated that UCMSCs facilitate collagen fiber degradation in uterine scars with concurrent upregulation of VEGF and downregulation of collagen I, as well as promoting the regeneration of the endometrium, myometrium, and blood vessels in uterine scars. These findings also support former reports that the expression of fibrotic markers CTGF, TGF-β, collagen I, and collagen III was increased in an IUA rat model but was decreased with MSC administration [7]. Previously, the development of fibrosis in irradiated lungs was also reported to be restricted after the administration of MSCs through an attenuation of the immunoexpression of collagen I and collagen III [26].

Likewise, the present results revealed a significant reduction in IL-1 mRNA expression in group IV (IUAs + hUCMSCs-EVs group) compared with that in group II (IUAs group). IL-1 plays a crucial role in the development of liver fibrosis and cirrhosis by enhancing hepatic stellate cell (HSC) production [48]. Similarly, the infusion of bone marrow-derived MSCs diminished skin thickening, contracture, collagen deposition, and decreased the expression of IL-1 in irradiated skin [49]. Similarly, a significant reduction in IL-6 expression in group IV (IUAs + hUCMSCs-EVs) compared with the expression in groups II (IUAs) and III (IUAs + estrogen) was noted. IL-6 has been implicated in the repair of compromised tissue by shifting the acute inflammation into a chronic profibrotic state. In accordance with the present findings, UCMSC administration decreased inflammation and inhibited the expression of IL-1, IL-6, TNF, collagen I, and TGF-β in bleomycin-induced lung fibrosis in mice [50]. Collectively, the hUCMSCs-EVs manifest their immunomodulatory effects through nonspecific and specific anti-inflammatory properties.

In group V (IUAs + estrogen + hUCMSCs-EVs), endometrial TGF-β was significantly reduced compared with that in group II (IUAs) (Fig. 4). This finding is in accordance with the positive association of the concentration of TGF-β with the intrauterine adhesion area. TGF-β is suspected to be involved in the reformation of intrauterine adhesions [51]. Similarly, another study suggested that hUCMSCs-EVs could enhance carbon tetrachloride (CCl_4_)-induced liver fibrosis through the reduction of TGF-β gene expression [52]. Additionally, in the present study, group V demonstrated the highest significant decrease in IL-6 expression of all the studied groups. In agreement with our results, IL-1, IL-6, IL-10, and TNF have been found to be significantly downregulated in a mare endometritis model with chronic pathological endometrial changes, including fibrosis, after mesenchymal stem cell treatment [53].

Notably, the endometrial stem cell marker CD140b was significantly upregulated in the hUCMSCs-EV-treated groups (groups IV and V). These results reflect the effect of hUCMSCs-EV treatment on directing the endometrial stem cell to differentiate correctly into endometrium in order to regenerate the injured uterine tissue. Treatment with hUCMSCs-EVs improved the expression of inflammatory and fibrotic markers leading to an improved uterine environment. On the other hand, CD140b was also significantly upregulated in the IUAs group and the estrogen-treated group (groups II and III, respectively) in this study. However, this upregulation indicates the response of endometrial stem cells to a disrupted uterine microenvironment, which favors misdifferentiation into fibroblasts and fibrosis.

In addition, the present study revealed a significant increase in VEGF gene expression in uterine tissues in groups IV and V compared with the expression in group II. In this context, our data are in accordance with earlier results showing that VEGF mRNA was upregulated in endometrial fibrosis rats treated with hUCMSCs-EVs alone and hUCMSCs-EVs combined with estrogen. Angiogenesis and hypoxic changes in the endometrial glands and interstitium were also improved in endometrial fibrosis patients treated with hormonal therapy [51].

RUNX2 expression in group IV and group V was significantly increased compared with the expression in group II (IUAs group). RUNX2 is a unique target for defense against fibrosis-related diseases. Previous results have also suggested that RUNX2 controls the progression of kidney fibrosis, in part through inhibition of the TGF-β signal [54]. Importantly, RUNX2 has been found to be essential for the blockade of TGF-β-driven fibroblast proliferation and differentiation into myofibroblasts, i.e., EMT [55]. Such a role was recently further supported by the results of Mümmler et al. [56] who attributed lung fibrosis and myofibroblast differentiation to RUNX2 downregulation in primary human lung fibroblasts. However, the authors also reported that RUNX2 mRNA was significantly increased in a whole-lung homogenate from idiopathic pulmonary fibrosis patients/pleomycin-lung fibrosis rat models compared with its expression in the relevant controls. Interestingly, differential expression of RUNX2 in pulmonary cells (i.e., a significant increase in alveolar-type epithelial cells coupled with a significant decrease in fibroblasts) has been documented. Although these observations are contradictory to ours, the discrepancy might be explained by differences in the organs studied or different methods for the induction of fibrosis.

## Conclusion

The results of this study suggest that estrogen, which is the present treatment used to rebuild the endometrium in cases of Asherman’s syndrome, is not a sufficiently effective therapy. Additionally, the combination of estrogen and hUCMSCs-EVs for the treatment of IUAs can regenerate the damaged endometrium and reverse fibrosis. Additionally, hUCMSCs-EVs encourage estrogen function that re-establishes endometrial function by improving the endometrial microenvironment. This function of hUCMSCs-EVs might explain why the best effect was observed in the group treated with the combination of hUCMSCs-EVs and estrogen. Therefore, to a certain extent, stem cell-derived EVs potentially represent a relevant therapeutic option for the treatment of IUAs instead of stem cells alone. On the other hand, the major limitation of this study was the inability to conduct a fertility assessment to evaluate the potential fertility improvements. Therefore, we could not investigate later stages, and further study is needed to evaluate their differential fertility potential which is the gold standard for the management of those suffering from infertility.
